# Human Left Ventral Premotor Cortex Mediates Matching of Hand Posture to Object Use

**DOI:** 10.1371/journal.pone.0070480

**Published:** 2013-07-30

**Authors:** Guy Vingerhoets, Jo Nys, Pieterjan Honoré, Elisabeth Vandekerckhove, Pieter Vandemaele

**Affiliations:** 1 Department of Experimental Psychology, Ghent University, Ghent, Belgium; 2 Ghent Institute for Functional and Metabolic Imaging, Ghent University, Ghent, Belgium; 3 Department of Radiology, Ghent University, Ghent, Belgium; Weill Cornell Medical College, United States of America

## Abstract

Visuomotor transformations for grasping have been associated with a fronto-parietal network in the monkey brain. The human homologue of the parietal monkey region (AIP) has been identified as the anterior part of the intraparietal sulcus (aIPS), whereas the putative human equivalent of the monkey frontal region (F5) is located in the ventral part of the premotor cortex (vPMC). Results from animal studies suggest that monkey F5 is involved in the selection of appropriate hand postures relative to the constraints of the task. In humans, the functional roles of aIPS and vPMC appear to be more complex and the relative contribution of each region to grasp selection remains uncertain. The present study aimed to identify modulation in brain areas sensitive to the difficulty level of tool object - hand posture matching. Seventeen healthy right handed participants underwent fMRI while observing pictures of familiar tool objects followed by pictures of hand postures. The task was to decide whether the hand posture matched the functional use of the previously shown object. Conditions were manipulated for level of difficulty. Compared to a picture matching control task, the tool object – hand posture matching conditions conjointly showed increased modulation in several left hemispheric regions of the superior and inferior parietal lobules (including aIPS), the middle occipital gyrus, and the inferior temporal gyrus. Comparison of hard versus easy conditions selectively modulated the left inferior frontal gyrus with peak activity located in its opercular part (Brodmann area (BA) 44). We suggest that in the human brain, vPMC/BA44 is involved in the matching of hand posture configurations in accordance with visual and functional demands.

## Introduction

In humans, goal based object-related movements play a significant role in our every day lives. These complex movements are composed of several components such as the reach, the grasp, and the manipulation part of the action, that, in concert, will contribute to the desired goal directed movement. Evidence is accumulating that the neural network underlying transitive movements is very complex, and that different movement components may be subserved by different neural regions [1]–[4]. In this study we will focus on the grasp part of the action, more in particular on the selection of the proper hand posture to functionally interact with a tool object. We will try to determine the neural correlates involved in the matching process.

Successful grasping involves the transformation of intrinsic object properties into motor actions [5]. Visual inspection of the object’s characteristics (size, shape, weight, texture) as well as the object’s position (distance, angle) will activate the proper motor schemas and shape the hand posture for an adequate reach and grasp movement. In monkeys, visuomotor transformations for grasping have been associated with two key cortical areas: area F5 or the rostral part of the monkey ventral premotor cortex, and area AIP or the rostral part of the intraparietal sulcus [6]. Inactivation studies of both areas resulted in impaired shaping of the hand relative to the object’s size and shape [7], [8]. Based on the characteristics of neurons in F5 and AIP, Fagg and Arbib proposed a model in which AIP uses visual input to highlight object features that are relevant for grasping it, whereas area F5 serves to select the most appropriate grasp in function of relevant constraints (visual information, task information, instructions). This decision is then relayed back to the AIP which focuses on the selected grasp and continually reinforces its inputs while F5 governs the motor execution and monitors the planned preshape and grasp [9].

In the human brain, the putative homologue for the monkey AIP was determined as the anterior segment of the intraparietal sulcus, commonly termed aIPS. Binkofski et al. documented selective deficits in the coordination of finger movements during object grasping in patients with lesions involving the aIPS [10]. These observations have been corroborated by neuroimaging studies when healthy participants perform simple prehensile actions [2], [10]–[13]. But the human aIPS has also been associated with action planning, recognition of goal-directed hand-object movements, and motor semantics [14]–[19].

The putative human homologue for the monkey F5 area is identified as the pars opercularis, the posterior part of the inferior frontal gyrus, also described as the ventral premotor cortex (vPMC). More specifically, the pars opercularis appeared implicated during the imitation of goal-oriented actions [20], observation of realized prehensile actions [21], [22] and action sequences [23], and that it is able to code action content in an abstract modality-independent fashion [24].

The findings seem to suggest that the role of the human fronto-parietal grasping system may be more complicated than that of the monkey, as both regions in the human brain seem especially sensitive to the conceptual high-level components of the transitive action. Any comparison between transitive gestures in human and non-human primates should take into account the much higher complexity of hand-related functions in humans. Only recently, a systematic comparison between human and non-human primates on the cognitive capacities deemed crucial to tool use concluded that human tool use reflects a profound discontinuity between us and our closest relatives [25].

In order to differentiate the contribution of aIPS and vPMC several studies have focused on grasp selection which, according to the Fagg & Arbib-model, should be subserved by the vPMC region. Grèzes et al. aimed to identify regions that responded to different grasp observation and execution conditions in a paradigm that required the selection of a power grip or a precision grip [26]. They found that the left vPMC and inferior frontal gyrus (IFG) Brodmann area (BA) 44 were selectively modulated during gesture imitation and gesture execution in response to objects. Buxbaum et al. compared the neural response during the selection of prehensile and non-prehensile hand postures for functional object use, versus prehensile postures used for grasping [1]. Difficulty of the experimental conditions were equated in terms of accuracy and response time. Significantly greater activations were indeed reported in the left IFG, but also in the posterior superior temporal gyrus (pSTG) and inferior parietal lobule (IPL) in the non-prehensile use condition as compared to the (prehensile) grasp condition. No differences were reported between the prehensile use condition and the (prehensile) grasp condition, and comparison of the non-prehensile use condition and prehensile use condition revealed a difference in the left IPL only. Buxbaum and colleagues interpreted their data to confirm the left IPL as a repository of hand postures for functional use [1]. A recent study by Makuuchi et al. compared the neural correlates of mimed object grasping in which the volunteers used the same or different grip types in the second presentation of an identical object. In the ‘different’ condition, taken to reflect increased selection demands, involvement of the vPMC, aIPS, and posterior inferior temporal gyrus (pITG) was found. Subsequent effective connectivity analysis suggested to the authors that the vPMC integrates the neural information of different regions (including aIPS, pITG, and dorsolateral prefrontal cortex (DLPFC)) to select the hand posture [27].

Taken together, the latter studies suggest that if grip selection is part of the grasping task, involvement of the ventral premotor region is more likely, although additional posterior parietal activation, in particular around the aIPS, is frequently observed. As a result, different interpretations for the role of putative human AIP and F5 in grasp selection have been proposed.

**Figure 1:**
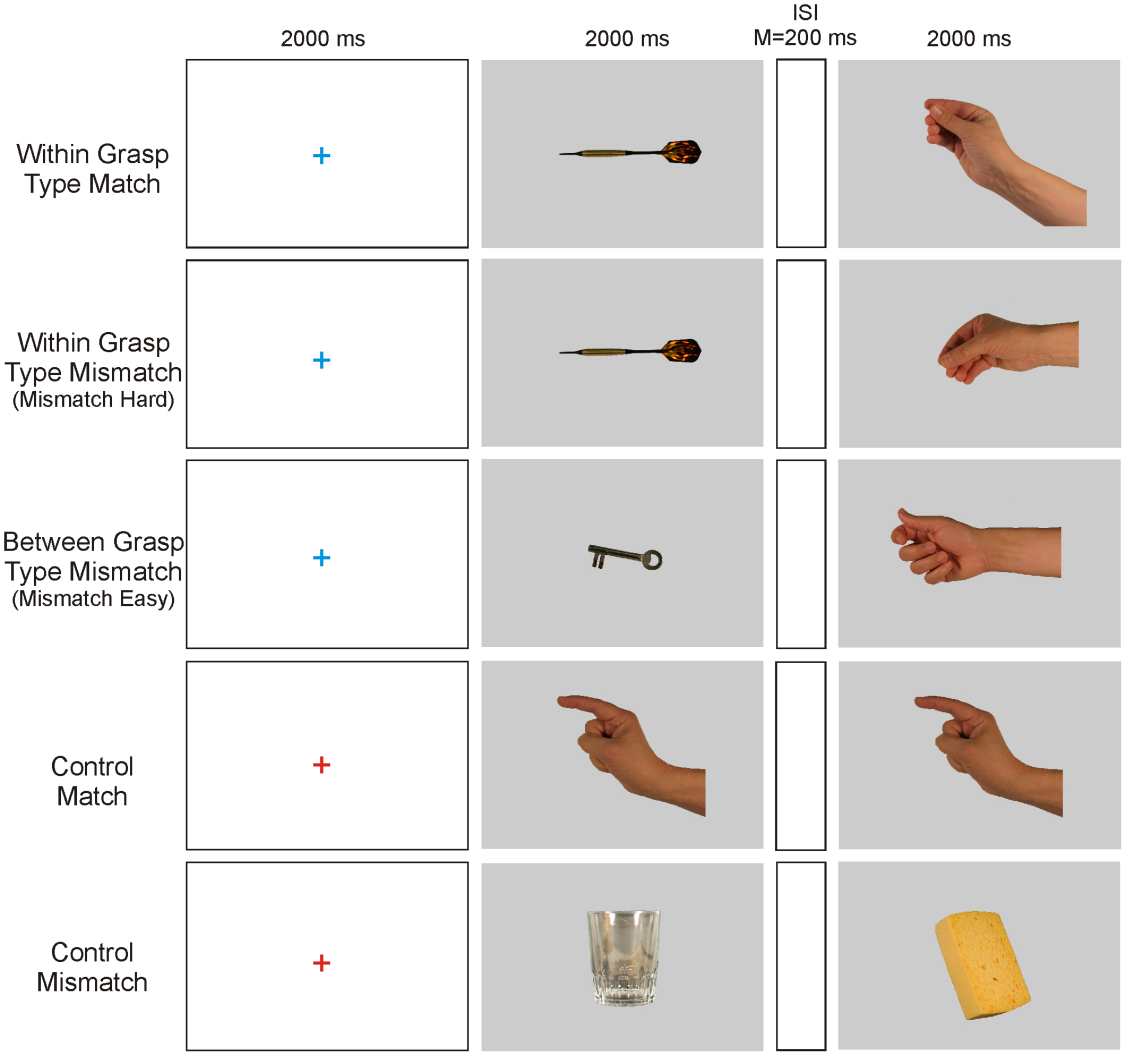
Structure of the paradigm and examples of the four conditions. In the experimental conditions, participants had do decide as quickly as possible whether the hand posture matched the functional use of a previously shown tool object (Match, Mismatch Easy, Mismatch Hard). In the Control condition, the volunteers had to decide whether both pictures were identical. ISI = Inter stimulus interval.

The aim of the present study was to focus on tool object – hand posture matching and to determine which brain areas would respond to increased matching demands. Volunteers were shown pictures of tool objects followed by pictures of hand postures that could match the functional use of the object or not (see Figure 1 for an overview of the paradigm, more details are provided in the Methods section). Their match/mismatch decision was registered with a button press. All presented stimuli were static and the task did not require actual or pantomimed grasping within the scanner environment, thus eliminating effects of motion and motor execution. The difficulty level of the experimental conditions was manipulated by selecting tool object - hand posture decisions between or within grip types in order to make the decision easy or hard. We hypothesized that conditions where demands on differentiation of hand posture and finger composition were higher, would show enhanced modulation in the neural region responsible for hand posture selection, and that this region would most likely correspond to the ventral premotor cortex.

## Results

### Behavioral Data

**Figure 2:**
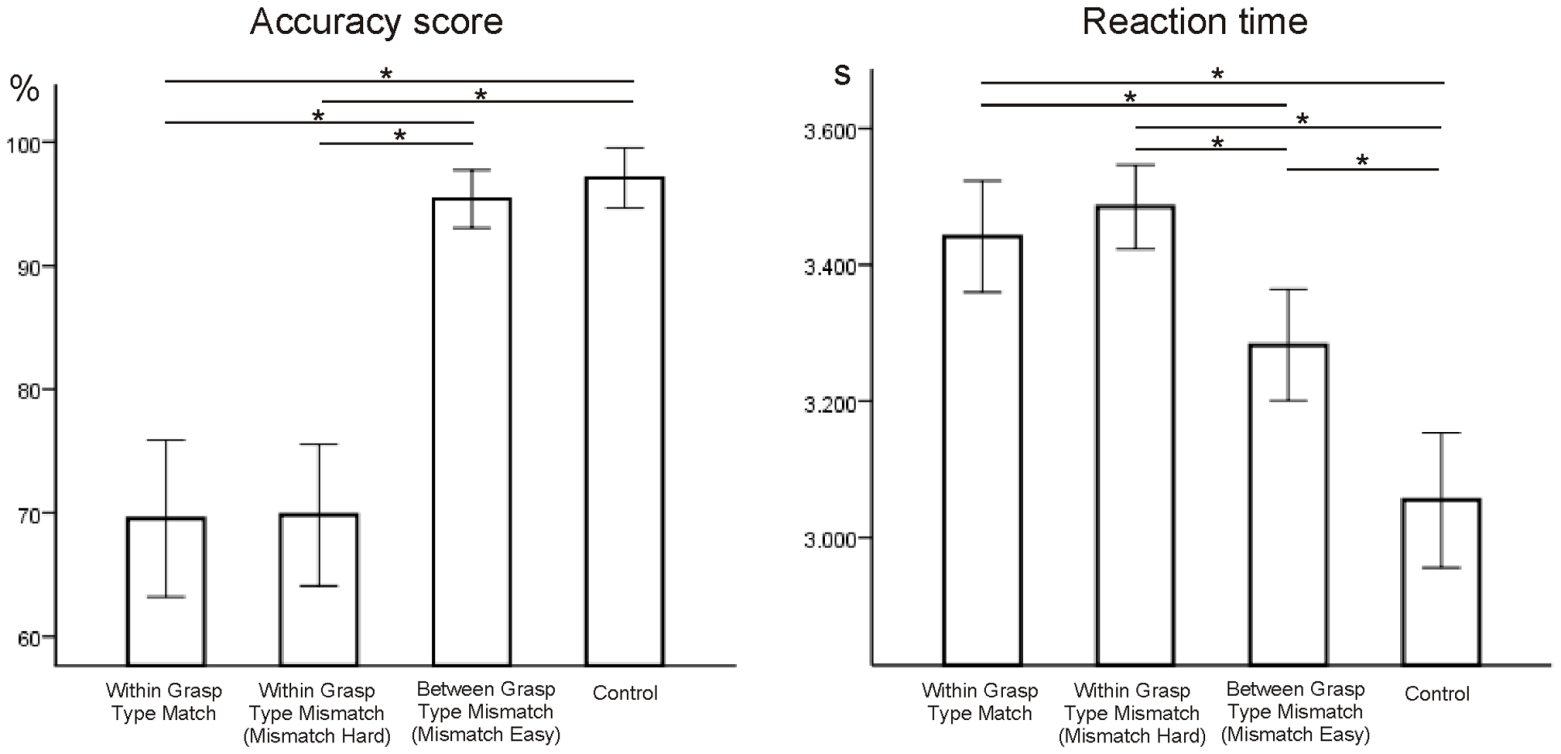
Behavioral performance. Left hand graph depicts percent accuracy scores in the four conditions. Right hand graph illustrates the conditions’ reaction times. Results of the post hoc paired-sample t-tests are indicated above the bars. * implies a p-value <.001. Error bars represent 95% confidence intervals of the mean.

A repeated measures analysis of variance on the *accuracy data* revealed a significant effect of condition, *F* [3], [14] = 161.97, *p*<.001. As illustrated in Figure 2, pairwise post-hoc comparisons indicated that performance accuracy of the Control and Mismatch Easy conditions differed significantly from the Match and Mismatch Hard conditions. No significant accuracy differences were found between Control and Mismatch Easy, and between Match and Mismatch Hard.

Similar statistics applied on *response times* (defined as time since first image of the sequence) also revealed a significant effect of condition, F[3], [14] = 30.53, p<.001. Post-hoc paired sample *t*-tests revealed significant differences between all conditions, except between Match and Mismatch Hard (Figure 2).

Importantly, these analyses confirmed a significant difference between the Mismatch Easy and Mismatch Hard conditions, with the latter showing an increased response time and reduced accuracy score. In addition, both Within grasp type choices (Match and Mismatch Hard) showed very similar accuracy and response speed data.

### Neuroimaging Data

**Figure 3:**
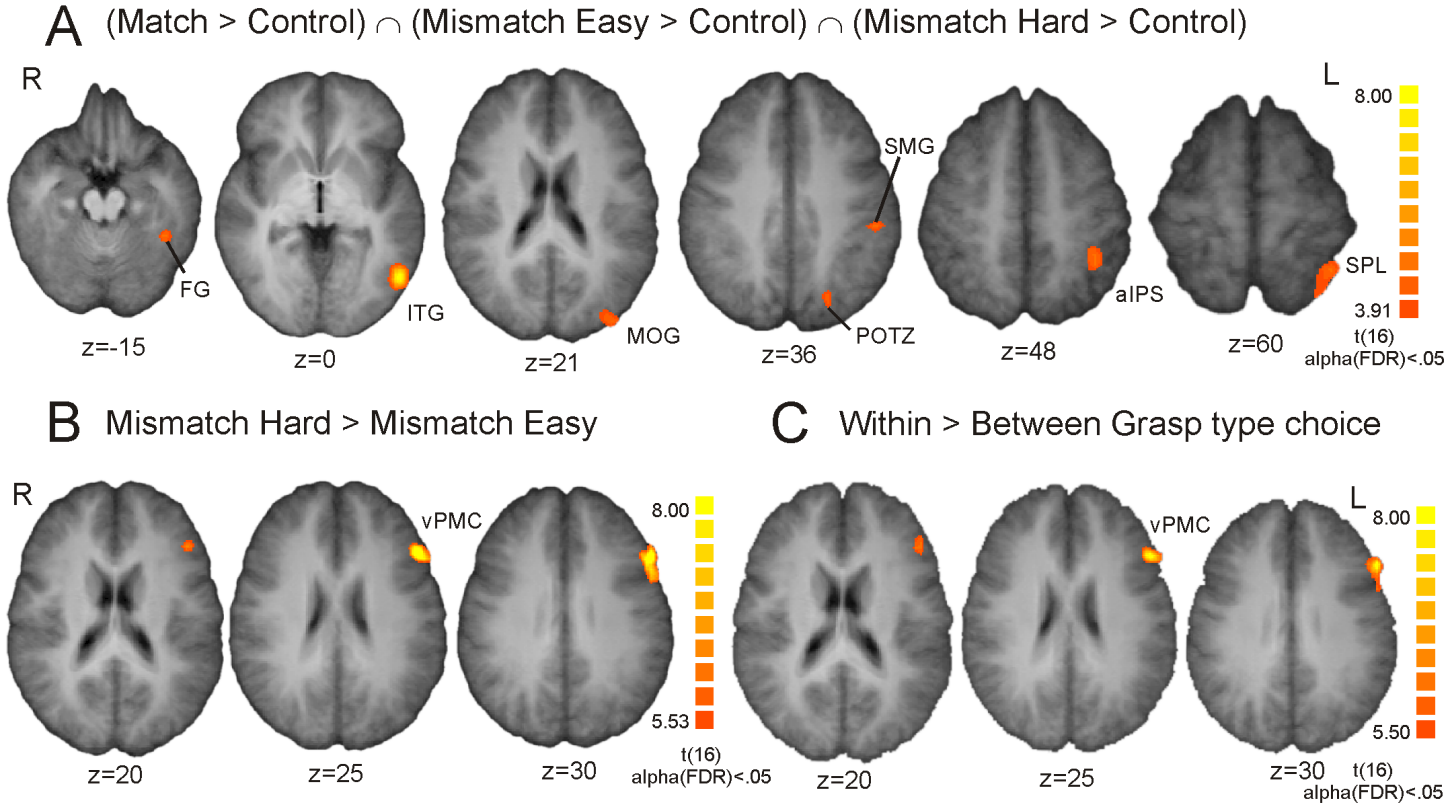
Group statistical maps for the contrasts of interest. A. Activation maps of the conjunction analysis comparing each experimental condition versus control at alpha(FDR)<0.05. B. Activation maps of the Mismatch Hard>Mismatch Easy contrast at alpha(FDR)<0.05. C. Activation maps of the Within>Between Grasp type choice conditions at alpha(FDR)<0.05. FG: fusiform gyrus; ITG: inferior temporal gyrus; MOG: middle occipital gyrus; POTZ: parieto-occipital transition zone; SMG: supramarginal gyrus; aIPS: anterior intraparietal sulcus; SPL: superior parietal lobule; vPMC: ventral premotor cortex.

The results of the conjunction of the experimental tasks compared to the picture matching control task are listed in Table 1 and depicted in Figure 3A. This conjunction analysis revealed a uniquely left lateralized occipito-temporo-parietal activation pattern. Posterior parietal activation was observed in the aIPS region, as well as in the supramarginal gyrus and the superior parietal lobule. Note that no frontal activation survived this contrast.

**Table 1:** Hand posture to object matching.

Brain region	BA	Talairach coordinates				Voxel count(1×1×1 mm)	t_max_
		X	Y		Z		
**(Match>Control) ∩ (Mismatch Easy>Control) ∩ (Mismatch Hard>Control)**							
Parietal clusters							
Inferior parietal lobule (SMG)^1^	40	−49	−29		36	108	5.05
Inferior parietal lobule (aIPS)	40	−37	−41		48	277	4.96
Superior parietal lobule	7	−34	−47		60	62	4.95
Parieto-occipital transition zone	7	−22	−71		36	63	4.74
Temporal clusters							
Fusiform gyrus	37	−40	−44		−15	87	4.99
Inferior temporal gyrus	37	−52	−65		0	1647	8.81
Occipital cluster							
Middle occipital gyrus	19	−37	−86		21	586	4.93
**Mismatch Hard>Mismatch Easy**							
Inferior frontal gyrus, opercular part	44	−52		19	27	1667	12.22
**Within>Between Grasp type choice (Match+Mismatch Hard >2 Mismatch Easy)**							
Inferior frontal gyrus, opercular part	44	−49		22	26	1226	9.11

1 SMG: supramarginal gyrus.

Coordinates of peak activity for the total group, alpha (FDR) <0.05.

Direct comparison of the hard versus the easy mismatch hand posture – object matching task revealed selective left hemispheric modulation over the inferior frontal gyrus. Peak activity in this cluster was found over the opercular part (Brodmann area 44; See Table 1 and Figure 3B). Contrasting both more difficult Within Grasp type choice conditions (Match and Mismatch Hard) with the Between Grasp type choice condition (Mismatch Easy) resulted in a similar activation pattern with peak activity in the frontal operculum, BA44 (See Table 1 and Figure 3C).

## Discussion

Compared to the picture matching control task, the experimental tool object – hand posture matching conditions jointly modulated several regions in the posterior part of the left hemisphere. This strong leftward activation during a task related to praxis in right handers is in agreement with neuropsychological and neuroimaging research [28]–[31].

As expected, increased modulation of the aIPS in the lateral bank (IPL) was obtained, and this region has repeatedly been implicated in prehensile movements and grasping intentions [2], [10]–[19], [32]–[34]. Inferior and lateral to this region, enhanced modulation in the supramarginal gyrus (SMG) was observed. This region on the convex portion of the inferior parietal lobule has been reported in paradigms comparing the observation and (imagined) manipulation of familiar as opposed to unfamiliar tools [18], [35]. The SMG is associated with ideomotor apraxia and is believed to store representations of the limb and hand subserving skilled object-related actions [36]–[39]. As our paradigm presented familiar objects and explicitly referred to hand postures necessary for their use, activation of this area is not unexpected. Two additional parietal regions, but now associated with a more dorsal position within BA 7, showed increased modulation in the experimental tasks. The first is positioned in the more anterior portion of the superior parietal lobule. Paradigms that activate this region are mainly concerned with shifts in spatial attention to moving targets [40]–[43], and in contrasts pertaining to the effect of perspective in action observation research [44], [45]. The second region is located more posterior and inferior within BA 7 and is described as a parieto-occipital transition zone (POTZ) in Mai et al. [46]. The junction between occipital and parietal cortex has been associated with severe misreaching in patients with so-called optic ataxia, a deficit in motor control characterized by poor and awkward reach trajectories and grasping of objects in the peripheral visual fields [47]. These neuropsychological findings have been tallied by neuroimaging studies showing that the activity in POTZ reflects coding of reach direction and the transport component of reaches [32], [48], [49]. Activation of both dorsal regions in this contrast seem to suggest that our participants may have imagined reaching for and grasping the presented objects in order to comply with the experimental tasks.

Extra-parietal modulation was unveiled in the left occipital (middle occipital gyrus, MOG) and temporal lobes (inferior temporal gyrus, ITG, and fusiform gyrus, FG). Neural activation caused by object stimuli is likely to be reflected in visual areas that are concerned with object recognition such as the fusiform cortex and the lateral occipital complex [14], [35], [50]–[52]. This would explain the activation in occipital and inferior temporal (including fusiform) regions in our volunteers. On the other hand, it can be argued that a similar kind and number of objects were used in the control condition. Why then would there be a higher modulation of these ventral regions in the experimental conditions? A possible explanation could be that in the experimental tasks the focus is not only on object identification, but also on the motor affordances of the depicted object, as the participant will have to compare the object’s structure against a hand posture. Research has shown that motor affordances are most readily determined by the object’s physical appearance, rather than by its conceptual information [53]. In addition, it has been suggested that the processing carried out in the fusiform gyrus may be more responsive to the object’s structure, than to its meaning [54]. If we combine these two lines of evidence, it becomes plausible to obtain elevated modulation during the experimental tasks, at least in the fusiform gyrus, because it is the object’s structure that conveys the most relevant information to solve the task. Note that in this conjunction analysis, no frontal activation, in particular of the vPMC, was encountered. This was mainly due to the fact that in the ‘Mismatch Easy>Control’ part of the conjunction no significant vPMC activation was obtained.

The behavioral data revealed a successful manipulation of the mismatch conditions’ difficulty level. Selecting a mismatch *between* posture types resulted in higher accuracy scores and faster response times than deciding on a mismatch *within* a hand posture type. In the Within Grasp type choice conditions the Match condition appeared to be equally difficult than the Mismatch Hard condition, as subtle differences within hand posture types had to be considered here too. Comparison of easy versus more difficult conditions was taken to reflect selective modulation in those brain areas that would have to deal with this increased task demand. In the Mismatch Hard>Mismatch Easy contrast, substantial response to task difficulty was elicited in the left ventral premotor cortex, in particular in pars opercularis (BA 44) of the inferior frontal gyrus. The same region was active in the more general Within>Between Grasp type choice contrast. These findings are in agreement with other studies that targeted the hand posture selection process and found vPMC activation among other activated regions. The merit of the present study is that it highlights the selective response of this region to differing demands in the discrimination of hand posture choice [26], [27], [37]. The selective involvement of vPMC in hand posture discrimination relative to object properties remains in agreement with the functional role of primate F5 as proposed by Fagg & Arbib [9], despite the increased complexity of transitive actions in humans. Rizzolatti et al. reported that of all the neurons active during grasping in the macaque’s F5 region, 85% were selective to specific types of prehension, the most frequent being a precision grip [55]. In humans, precision grips also revealed stronger modulation in the vPMC/BA 44 area (among other regions) compared to power grips, in particular when small grip forces rather than excessive grip forces were applied [56], [57]. In addition, it has been shown that the usual muscle-specific vPMC-PM interactions that appeared during grasp preparation were significantly reduced following aIPS perturbation (TMS), and that this disruption was behaviorally associated with a reduced grasp-specific pattern of digit muscle activity [58]. These findings and the results of the current study suggest that stronger demands on task-related muscular configurations, whether reflecting finger movement, hand posture, or fingertip force control appear to engage a primate’s ventral premotor cortex. Future research should determine this region’s selectivity for prehensile (as compared to non-prehensile) object-related gestures, provide more direct proof of a close relation between parametric variation in motor-muscular complexity (computational demand) and vPMC BOLD response, and ascertain whether these demands reveal multiple vPMC representations for separate transitive qualities such as posture, movement, or force.

## Methods

### Stimuli

Twenty-eight familiar tool objects were selected, a list of which can be found in Appendix S1. Functional use of these tools would require a power grip (n = 12), precision grip (n = 10), poke posture (n = 3), or palm posture (n = 3). Healthy participants can reliably associate these four hand postures to the use of objects and in daily life prehensile postures are more common than non-prehensile postures [59]. Each object was photographed in a comfortable right hand grasp position using a Canon EOS 300D digital reflex camera. The right handed experimenter (GV) then grasped the object in a functional manner, and carefully removed the object out of his grip while maintaining the hand posture for that particular object’s use. Again, a still picture of that hand posture was made. All object and hand posture stimuli were depicted on a neutral grey background. Based on these 28 static pictures of objects and their corresponding 28 functional hand positions, four conditions were created (Figure 1). For the first two conditions an object was paired with a hand posture that was compatible with its functional use (for example a dart with a precision grip), but could be either the proper precision grip for that particular object’s use (Match decision) or an incorrect precision grip (Mismatch decision). As these conditions require discriminations of compatible grasps, they are referred to as ‘Within Grasp type choice’. In both cases, the decision has to be based on a careful consideration of the correspondence between the object’s size, shape, and inclination with the precise hand posture (finger or clench aperture, hand inclination, etc.). These conditions are likely to be difficult, and this mismatch decision is referred to as the Mismatch Hard condition. In a third condition, each object was paired with a hand posture that belonged to a different hand posture category, and is referred to as ‘Between Grasp type choice’. For example by combining a key (precision grip) with a power grip posture. In this condition the mismatch between object and hand posture was relatively easy to determine, and this condition was described as Mismatch Easy. Finally, in a Control condition, an object or a hand posture image was paired with either the same or a different object or hand posture picture respectively. In this condition, we coupled 14 object-object pairs and 14 hand-hand pairs, so that the visual input was identical in all conditions. Thus four sets of 28 stimulus pairs were created that made up the four conditions of the experiment.

### Participants

Seventeen healthy volunteers participated in the study (age range: 20−40 years, mean age: 23.3; 11 women and 6 men). All were right-handed as determined by the Edinburgh Handedness Inventory: *M* = 93.6%, *SD* = 8.8% [60] and none had a history of neurological or psychiatric disease. Scanning protocols were approved by the Ethics Committee of the University Hospital Ghent and all subjects gave written informed consent after the experimental procedure had been explained to them.

### Procedure

Prior to scanning, the volunteers completed a pre-scan MRI-safety questionnaire and the Edinburgh Handedness Inventory. They were instructed that each experimental trial would start with a blue fixation cross. After the fixation cross, they were going to see a picture of a tool object followed by a picture of a hand posture, and they would have to decide as quickly as possible whether the hand posture shown corresponded to the functional use of the previously presented object. If the trial started with a red fixation cross, the pictures could depict two consecutive objects or hand postures. In that case they would have to decide whether both images were identical or different. If they decided that the hand posture matched the functional use of the paired object, or if both stimuli were the same, they had to press the right button of an MR compatible button press with their left index finger. If they felt that the hand posture did not match this object’s use, or if both pictures depicted different objects or hand postures, they had to press the left button with their left middle finger. We made it clear that accuracy was more important than speed, but once decided, a timely response had to be made.

The volunteers were positioned head first and supine in the magnet with their left and right arms placed alongside the body on the scanner table. The button press was placed on the scanner table under the left hand and was controlled with the middle and index fingers. Participants were reminded of the fact that MR-imaging is very sensitive to movement and were required to restrict head movements and to lie as still as possible in order to prevent motion artifacts. Their heads were gently fixed in place with foam cushions and stimuli were presented through goggles with an MRI-compatible presentation system (VisuaStim-Digital, Resonance Technology Inc., California, USA).

Stimulus presentation and response recording was controlled by a commercially available experiment generator (Presentation, Neurobehavioral Systems Inc., Albany CA, USA). Each trial started with a 2000 ms fixation cross (blue cross: tool object - hand posture match; red cross: picture match). Next, the first picture of the stimulus pair appeared on the screen for 2000 ms, followed by a variable interval (mean interval time = 200 ms). After the interval the second picture appeared for 2000 ms. Each stimulus pair was shown twice: 4 conditions× (2×28 stimulus pairs) = 224 trials. The paradigm was arranged as a permuted block-design with four conditions: Match, Mismatch Easy, Mismatch Hard, and Control. A permuted block design was chosen to avoid psychological confounds associated with traditional block designs, such as habituation and anticipation. In comparison with event related designs, permuted block designs also obtain advantageous trade-offs between efficiency, detection power, and conditional entropy or randomness. Permutation was achieved by exchanging the positions of two randomly chosen events in a classic block design, that with each iteration (n = 100) became increasingly random [61]. In total, the experiment took 23 minutes (224 trials of 6200 ms each), and stimuli were randomly distributed over their conditions’ (permuted) blocks. In the post-scan session, participants completed a post-scan MRI safety questionnaire and were debriefed.

### Data Acquisition

Scanning was performed at 3.0 T on a Siemens Trio MRI scanner (Siemens Medical Systems, Erlangen, Germany) that was equipped with echo planar imaging (EPI) capabilities and used an 8-channel PA head coil for radio frequency transmission and signal reception. After automatic shimming of the magnetic field on each participant, a 3-D high-resolution T 1 anatomical image of the whole brain in the sagittal plane was acquired for coregistration with the functional images (3D MPRAGE, 176 slices, slice thickness = 0.9, in-plane resolution = 0.9×0.9 mm, TR = 2530 ms, TE = 2.58). Next, 560 functional EPI images in the axial plane were acquired for the matching paradigm with the following parameters: TR = 2.5 s, TE = 33 ms; flip angle = 90°, 33 slices, slice thickness = 2.5 mm, slice gap = 1.25 mm, FOV = 192 mm and matrix = 64×64, resulting in a resolution of 3×3×2.5 mm.

### Image Analysis

Data analysis was performed using Brain Voyager QX for preprocessing and statistical inference [62]. Functional data were subjected to a standard sequence of preprocessing steps comprising slice scan time correction by means of sinc interpolation, 3-D motion correction by spatial alignment to the first volume also by means of sinc interpolation, and temporal filtering using linear trend removal and high pass filtering for low-frequency drifts of 3 or fewer cycles. Spatial smoothing with a Gaussian filter (FWHM = 8 mm) was applied for the volume-based analysis. The anatomical data for each subject were resampled to a 1×1×1 mm resolution. Transformation into Talairach standard space was performed in two steps. In the first step, the cerebrum is translated and rotated into the AC-PC plane (AC = anterior commissure, PC = posterior commissure). In the second step, the borders of the cerebrum are identified; in addition with the AC and PC points, the size of the brain is fitted into standard space. We used sinc interpolation as the transformation method as it applies no implicit smoothing. The functional data for each subject were coregistered with the subject’s 3-D anatomical dataset and transformed into Talairach space. After coregistration, a volume time course of the functional data was created and resampled into a cubic voxel of 3×3×3 mm.

For each subject’s paradigm, a protocol file was derived representing the period from the onset of the stimulus until the participant’s response for each trial of the different conditions. Factorial design matrices were automatically defined from the created protocols. The BOLD response in each condition was modeled by convolving these neural functions with a canonical hemodynamic response function (gamma) to form covariates in a General Linear Model (GLM). After the GLM had been fitted and the effects of temporal serial correlation allowed for (using AR(1) modeling, see [63]), group (random effects procedure) *t*-maps were generated to evaluate the effects of hand posture – object matching. First, we determined the general effect of hand posture selection by performing a conjunction analysis of all posture-object match conditions compared to the control (picture match) condition: (Match>Control) ∩ (Mismatch Easy>Control) ∩ (Mismatch Hard>Control). This conjunction analysis was executed on a whole-brain analysis. Second, we directly contrasted the easy and hard mismatch conditions to determine the neural correlates of more demanding hand posture selection: Mismatch Hard>Mismatch Easy. The comparison between Mismatch Hard and Mismatch Easy is the most straightforward comparison as both conditions require exactly the same response, namely a negative decision (mismatch) followed by a left middle finger press. We also performed the more general contrast of Within>Between Grasp type choice, namely Match+Mismatch Hard >2 Mismatch Easy. Indeed, the behavioral data revealed a similar difficulty level for the Match and the Mismatch Hard conditions (see below), which is not unexpected given that both conditions reflect a within grasp type decision. For all analyses, we used a threshold of *p*<.05 corrected for multiple comparisons using False Discovery Rate (FDR) correction [64]. Areas of significant activation were identified using the brain atlases of Mai et al. [46] and Talairach and Tournoux [65].

## Supporting Information

Appendix S1 **List of the familiar tool objects used in the paradigm.**
(DOC)
