# Supplementary material for: Human Left Ventral Premotor Cortex Mediates Matching of Hand Posture to Object Use
Source: PLoS One. 2013 Jul 30;8(7):e70480. doi: 10.1371/journal.pone.0070480 (PMC3728237; doi:10.1371/journal.pone.0070480)
Supplement: Appendix S1 — List of the familiar tool objects used in the paradigm. (DOC) [file pone.0070480.s001.doc]

Appendix 1

List of familiar tools used in the paradigm:

Lighter (power grip), ball pen (precision grip), calculator (poke posture), door bell (poke posture), drinking glass (power grip), eraser (precision grip), hair brush (power grip), hammer (power grip), nail brush (precision grip), telephone dial (rotary) (poke posture), coffee cup (precision grip), butter knife (power grip), needle (precision grip), bottle opener (power grip), paint brush (power grip), scissors (power grip), screwdriver (power grip), house key (precision grip), sponge (palm posture), teeth brush (power grip), telephone handset (power grip), brush (precision grip), dart (precision grip), face cloth (palm posture), clothes pin (precision grip), saw (power grip), shammy cloth (palm posture), salt shaker (precision grip).
